# AUY922 improves sensitivity to sunitinib in clear cell renal cell carcinoma based on network pharmacology and in vitro experiments

**DOI:** 10.1016/j.heliyon.2024.e34834

**Published:** 2024-07-18

**Authors:** Zixuan Chen, Xing Jia, Yuesong Cai, Ya Song, Yanjun Tong, Sheng Cheng, Min Liu

**Affiliations:** a Department of Urology, Tongren Hospital Shanghai Jiao Tong University School of Medicine, Shanghai, 200336, China; b Hongqiao International Institute of Medicine, Tongren Hospital Shanghai Jiao Tong University School of Medicine, Shanghai, 200336, China; c College of Medicine, Yanbian University, Yanji, 133002, China; d School of Life Sciences, Bengbu Medical University, Bengbu, 233000, China; e Department of Anesthesiology and Surgery, Tongren Hospital Shanghai Jiao Tong University School of Medicine, Shanghai, 200336, China

**Keywords:** HSP90B1, AUY922, Clear cell renal cell carcinoma, Sunitinib, Network pharmacology

## Abstract

Clear Cell Renal Cell Carcinoma (ccRCC), the most prevalent form of renal cell carcinoma (RCC), poses a significant threat to human health due to its rising morbidity and mortality rates. Sunitinib, a pivotal targeted drug for the treatment of ccRCC, presents a significant challenge due to the high susceptibility of ccRCC to resistance. HSP90 inhibitor AUY922 has demonstrated anti-tumor activity in a range of cancer types. However, its efficacy in combination with sunitinib for ccRCC treatment has not been evaluated. In this study, we employed bioinformatics, network pharmacology, and in vitro assays to verify that AUY922 inhibits cell viability, proliferation, and migration of ccRCC cell lines 786-O and ACHN, with IC50s of 91.86 μM for 786-O and 115.5 μM for ACHN. The effect of AUY922 enhancing the inhibitory effect of sunitinib on ccRCC was further confirmed. The CCK-8 assay demonstrated that the IC50 of sunitinib was reduced from 15.10 μM to 11.91 μM for 786-O and from 17.65 μM to 13.66 μM for ACHN, after the combined application of AUY922. The EdU assay and wound healing assay indicated that AUY922 augmented the inhibitory impact of sunitinib on the proliferation and migration of ccRCC cells. Western blot and RT-PCR analyses demonstrated that AUY922 increased the sensitivity of ccRCC cells to sunitinib by targeting the HIF-1α/VEGFA/VEGFR pathway. Our study represents the first investigation into the role and mechanism of AUY922 in enhancing the sensitivity of ccRCC to sunitinib. In conclusion, the findings indicate the potential for AUY922 to enhance the therapeutic efficacy of sunitinib and overcome sunitinib resistance in ccRCC.

## 1 Introduction

Renal cell carcinoma (RCC) is the most prevalent type of kidney cancer, accounting for over 90 % of all kidney cancers. According to the International Agency for Research on Cancer [1], in 2022, approximately 430,000 patients worldwide were diagnosed with RCC, representing 2.2 % of all cancers, and approximately 150,000 patients died from RCC. In China, there are approximately 70,000 new cases of RCC and 20,000 deaths from RCC annually. RCC poses a significant threat to human health due to its high incidence and mortality rates. RCC originates from renal tubular epithelial cells and is classified into several subtypes, with Clear Cell Renal Cell Carcinoma (ccRCC) being the most common type [2]. Currently, surgical treatment options such as radical or partial nephrectomy offer the possibility of a cure for most ccRCC patients. However, ccRCC is prone to recurrence and metastasis, which limits the effectiveness of surgical treatments. Fortunately, the advent of anti-angiogenic drugs targeting vascular endothelial growth factor (VEGF) has led to significant progress in the treatment of ccRCC [3]. Sunitinib, a receptor tyrosine kinase (RTK) inhibitor, plays a pivotal role in the RCC treatment due to its ability to target various receptors, including vascular endothelial growth factor receptor (VEGFR) and platelet-derived growth factor receptors (PDGFR), thereby inhibiting angiogenesis and ultimately tumor growth [4]. Unfortunately, most ccRCC patients develop resistance to sunitinib within six months of its application, posing a serious challenge [5]. Consequently, the identification of novel agents that enhance the sensitivity of ccRCC to sunitinib, thereby improving the efficacy of sunitinib in ccRCC, has become a pressing matter requiring urgent investigation.

The human body contains various heat shock proteins (HSPs), with Heat Shock Protein 90 Beta Family Member 1 (HSP90B1) being one of the most widely distributed and highly conserved. Currently, it is observed that HSP90B1 is highly expressed under stress conditions, such as the presence of oncogenic factors [6]. HSP90B1 is typically located in the endoplasmic reticulum, where it inhibits apoptosis and autophagy by stabilizing and refolding proteins to ensure cell survival. However, in response to oncogenic factors, HSP90B1 is expressed at elevated levels in cancer cells, thereby enhancing their survival capacity and ultimately contributing to cancer initiation and progression [7]. Wang et al. [8] conducted a pan-cancer analysis and observed a significant upregulation of HSP90B1 in various cancer types, including RCC, which correlated with unfavorable prognoses. Consequently, targeting HSP90B1 may prove effective in diagnosing and treating cancer [9].

AUY922, a novel HSP90 inhibitor, has been validated as an effective therapeutic agent against various tumors, including hepatocellular carcinoma [10], non-small cell lung cancers [11], and colon cancer [12], among others. In the context of renal cancer, Zhu et al. [13] confirmed that AUY922 inhibits the proliferation of renal cancer cells. Beyond its tumor-suppressive effects, AUY922 has gained attention as an adjunctive medication that synergizes with chemotherapy agents to impede tumorigenesis [14]. However, no studies have yet examined the combined use of AUY922 and sunitinib to enhance the therapeutic efficacy in renal cancer treatment.

The aims of the study were to investigate the mechanism by which AUY922 enhances the sensitivity of ccRCC to sunitinib. The potential of AUY922 to improve the sensitivity of ccRCC to sunitinib was predicted through bioinformatics and network pharmacology analyses. The mechanism by which AUY922 enhances the therapeutic efficacy of sunitinib in ccRCC was verified through in vitro experiments. This study identified a novel approach for combining drugs to enhance the efficacy of sunitinib in ccRCC and provided a new therapeutic strategy to address the issue of sunitinib resistance in ccRCC.

## 2 Materials and methods

### 2.1 Sample collection

For this study, cancerous and adjacent non-cancerous tissue samples from seven ccRCC patients were collected from Tongren Hospital, Shanghai Jiao Tong University School of Medicine. All patients provided informed after being fully informed about the study. The study protocol received approval from the Ethics Committee of Tongren Hospital, Shanghai Jiao Tong University School of Medicine.

### 2.2 Bioinformatics analysis

Pan-cancer analysis of TCGA cohorts was conducted using the OmicShare tool (https://www.omicshare.com/tools). To verify the upregulation of HSP90B1 in tumor tissues, a total of 20 normal tissue samples and 20 ccRCC samples were obtained from the GEO dataset (GSE213324) and analyzed using GEO2R (https://www.ncbi.nlm.nih.gov/geo/geo2r/). This analysis compared the expression levels of HSP90B1 in normal tissues and ccRCC tissues. Gene expression analysis of TCGA and GTEx datasets was performed using the GEPIA2 online tool (http://gepia2.cancer-pku.cn/).

Clinical and follow-up data from the TCGA database were downloaded from the GDC portal (https://portal.gdc.cancer.gov/) for further analysis using R software. We analyzed the relationship between HSP90B1 expression levels and various clinicopathological characteristics, including WHO stage, clinical T stage, clinical N stage, and clinical M stage. The results of these analyses were visualized using the “ggplot2” package in R Language. TNM stage differential comparison test was performed using UALCAN online tool (https://ualcan.path.uab.edu/index.html) [15].

### 2.3 Network pharmacology analysis

A total of 100 target genes of AUY922 were identified using SwissTargetPrediction (http://swisstargetprediction.ch/) [16]. From the GeneCards database (https://www.genecards.org/), we obtained 1937 disease genes associated with ccRCC, selecting the top 200 genes for subsequent analysis based on the Relevance score, a numerical value representing gene-disease associations calculated using the Elasticsearch 7.11 platform. The overlapping genes between AUY922 target genes and ccRCC disease genes were analyzed using the bioinformatics (https://www.bioinformatics.com.cn/). Protein-protein interaction (PPI) networks of overlapping genes were constructed using STRING (https://cn.string-db.org/). These overlapping genes were then uploaded to the DAVID website (https://david.ncifcrf.gov/) [17], with “official gene symbol” selected for Identifier and “*Homo sapiens*” for Species. We conducted Gene Ontology (GO) analysis and Kyoto Encyclopedia of Genes and Genomes (KEGG) pathway analysis. The top six results, based on P value (P < 0.05), were visualized using the R language.

### 2.4 Cell lines

The normal human renal epithelial cell line HK-2, and the human ccRCC cell lines Caki-1, A498, 786-O and ACHN were obtained from Shanghai Zhong Qiao Xin Zhou Biotechnology (Shanghai, China). These cells were cultured in RPMI 1640 complete medium (ZQXZbio, China) or EMEM complete medium (ZQXZbio, China). The cultures were maintained in a humidified incubator with 5 % CO_2_ at 37 °C. For drug treatments, sunitinib (B1045, APExBIO, Houston, USA) and AUY922 (A4057, APExBIO, Houston, USA) were diluted to specific concentrations with complete medium, and the cell cultures were incubated with the drug mixture.

### 2.5 CCK-8 assay

786-O and ACHN were seeded into 96-well plates. After 24 h, the cells were treated with sunitinib, AUY922 or a combination of both. Following a 24-h drug treatment, the CCK-8 reagent (SB-CCK8, Share-bio, Shang Hai) was added to each well, and the plates were incubated for 1 h at 37 °C. Absorbance readings at 450 nm were obtained for each well using the Multiskan FC Microplate Reader (Thermo Scientific, USA). IC50 values were calculated using GraphPad Prism.

### 2.6 EdU assay

786-O and ACHN were seeded into 6-well plates and then treated using EdU Imaging Kits (K2240, APExBIO, Houston, USA). The specific procedure is as follows: cells were treated with drugs for 24 h. Subsequently, EdU was added, and the cells were incubated for 2 h at 37 °C. The cells were then fixed with paraformaldehyde for 15 min, followed by permeabilization with PBS containing 0.3 % Triton X-100 for 15 min. Next, the Click reaction mixture was prepared according to the protocol and incubated with the cells for 30 min in the dark. The nuclei were stained with Hoechst 33342 for 10 min. The staining was then observed using a confocal microscope. The proportion of EdU-positive cells were analyzed using ImageJ software.

### 2.7 Wound healing assay

786-O and ACHN were seeded into 6-well plates. After 48 h, a sterile 200 μL pipette tip was used to scratch the cell layer, and any floating cells were subsequently removed by washing with PBS. The cells were then cultured in complete medium containing the drugs. Images of would area were recorded at 0 and 24 h after scratching and were quantitatively analyzed using ImageJ software.

### 2.8 Weston blot

Cell lysates were prepared by treating the cells with RIPA lysis buffer (SB-BR040, Share-bio, Shang Hai), supplemented with a protease inhibitor cocktail and a phosphatase inhibitor cocktail (EpiZyme, China). The protein concentration was quantified using the BCA Protein Assay Kit (SB-WB013, Share-bio, Shang Hai). Proteins were separated by SDS-PAGE and subsequently transferred to PVDF membranes (Millipore Corporation, USA). The membranes were blocked with protein-free rapid blocking buffer (Epi-Zyme, China) for 20 min at room temperature. Specific primary antibodies were used to incubate the membranes overnight at 4 °C. The primary antibodies included GAPDH (1:10000, ab9485, Abcam), β-Tubulin (1:10000, ab15568, Abcam), VEGFA (1:1000, 66828-1-Ig, Proteintech), HIF-1α(1:1000, 20960-1-AP, Proteintech), VEGFR1 (1:1000, 13687-1-AP, Proteintech), VEGFR2 (1:1000, 26415-1-AP, Proteintech), HSP90B1(1:1000, 14700-1-AP, Proteintech). The membranes were washed three times with TBST and then probed with HRP-conjugated secondary antibodies (1:2000, 7074 S/7076 S, CST) for 1 h at room temperature. The blots were washed with TBST again. Visualization of the blots was performed using a Tanon-5200 chemiluminescence imaging system (Tanon Science & Technology, Shanghai, China), and gray-scale analysis was carried out using ImageJ software.

### 2.9 Real-time PCR (RT-PCR) analysis

Total RNA was extracted from the cells using RNA Quick Purification Kit (SB-R001, Share-bio, Shang Hai). Reverse transcription was performed with HiScript III RT SuperMix for qPCR (+gDNA wiper) (Vazyme, China) to obtain cDNA. RT-PCR were then conducted using ChamQ Universal SYBR qPCR Master Mix (Vazyme, China) and CFX96 Touch RT-PCR Detection System (Bio-Rad). The primers were designed at GENEWIZ (Suzhou, China). The primers are listed in Supplementary Table 1.

### 2.10 Statistical analysis

All experiments, unless otherwise indicated, were performed at least three times. Statistical analysis was conducted using GraphPad Prism 9.0 software. A one-way analysis of variance (ANOVA) was employed to ascertain the statistical significance of the observed differences between the treatment groups. Data are presented as mean ± SD; *P < 0.05, **P < 0.01, ***P < 0.001 and ****P < 0.0001.

## 3 Results

### 3.1 The expression of HSP90B1 in ccRCC in databases

**Fig. 1:**
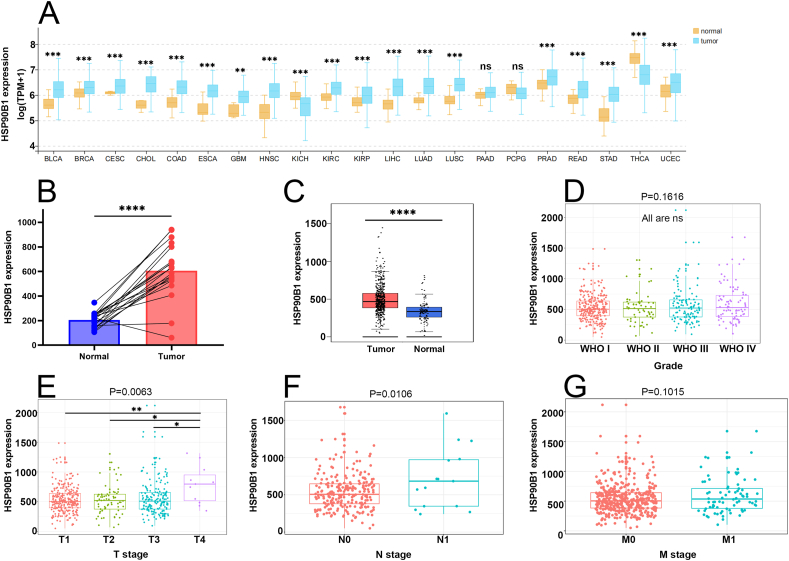
The expression of HSP90B1 in ccRCC in databases. (A) Pan-cancer analysis of the expression of HSP90B1 in TCGA database; (B) expression levels of the HSP90B1 between ccRCC tissues and their corresponding normal kidney tissue in GEO dataset; (C) expression levels of the HSP90B1 between ccRCC tissues and their corresponding normal kidney tissue in GEPIA2; Association between HSP90B1 expression and clinicopathologic characteristics, including (D) WHO Grade, (E) T stage, (F) N stage, (G) M stage.

We first analyzed the expression levels of HSP90B1 in clear cell renal cell carcinoma (ccRCC) using bioinformatics approaches. A pan-cancer analysis was conducted to explore the expression levels of HSP90B1 across various cancer types. The results indicated that HSP90B1 is highly expressed in multiple cancers, including bladder cancer (BLCA), prostate adenocarcinoma (PRAD), stomach adenocarcinoma (STAD), and kidney renal clear cell carcinoma (KIRC) (Fig. 1A). To validate these findings, we further analyzed the expression differences of HSP90B1 between ccRCC and normal tissues using the GEO2R and GEPIA websites. Analysis of 40 samples from the GEO database (excluding one sample lacking corresponding normal tissue data) with GEO2R showed that HSP90B1 expression was significantly higher in ccRCC tissues compared to normal tissues (p < 0.0001) (Fig. 1B). Consistent results were obtained from the GEPIA analysis, which utilized data from the TCGA and GTEx databases, indicating that HSP90B1 expression is significantly higher in ccRCC tissues than in normal tissues (p < 0.0001) (Fig. 1C).

Subsequently, we analyzed the relationship between HSP90B1 expression levels and clinicopathological features of ccRCC using 529 ccRCC samples obtained from the TCGA database. The results indicated that high expression of HSP90B1 is associated with T4 and N1 stages but not with WHO staging or M stage (Fig. 1D–G). It is well known that higher T and N stages indicate a greater likelihood of tumor metastasis [18], whereas the M stage signifies that metastasis has already occurred, and WHO staging is not related to tumor metastasis. Further analysis of HSP90B1 expression in normal tissues compared to ccRCC tissues at different TNM stages revealed that HSP90B1 expression levels are higher in ccRCC tissues with higher TNM stages compared to normal tissues (Table 1). These findings suggest that higher HSP90B1 expression in ccRCC patients may predict an increased risk of tumor metastasis and progression.

**Table 1:** Expression of HSP90B1 in ccRCC based on TNM stage.

Comparation	p value
Normal vs Stage 1	3.0521E-06
Normal vs Stage 2	9.1923E-04
Normal vs Stage 3	6.5373E-07
Normal vs Stage 4	4.4157E-08
Stage 1 vs Stage 2	0.3706
Stage 1 vs Stage 3	3.9842E-02
Stage 1 vs Stage 4	4.0015E-03
Stage 2 vs Stage 3	0.5365
Stage 2 vs Stage 4	0.1886
Stage 3 vs Stage 4	0.4426

### 3.2 HSP90B1 is highly expressed in ccRCC tissues and renal cancer cells

**Fig. 2:**
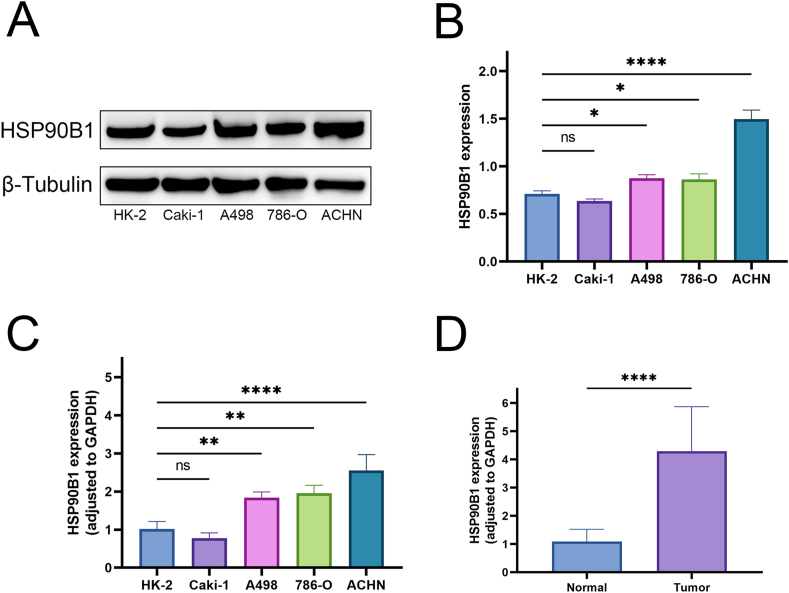
HSP90B1 is highly expressed in ccRCC tissues and renal cancer cells. (A) HSP90B1 expression in renal cancer cells was determined by Western Blot (The original image is provided in the Supplementary Fig. S1); (B) Quantification based on β-Tubulin served as internal reference of Western blot of HSP90B1 in cell lines; RT-PCR analysis of HSP90B1 ex-pression in (B) renal cancer cells and (C) ccRCC tissues based on GAPDH served as internal reference.

To enhance the credibility of our bioinformatics analysis, we validated the expression of HSP90B1 in both ccRCC tissues and renal cancer cells. Western blot analysis was employed to evaluate the levels of HSP90B1 protein expression. Comparing the normal human renal epithelial cell lines HK-2 to the human ccRCC cell lines Caki-1, 786-O, ACHN, and A498, the results revealed considerable overexpression of HSP90B1 in ccRCC cell lines, including 786-O, ACHN, and A498. while expression levels were not elevated in Caki-1 (Fig. 2A and B). RT-PCR analysis confirmed the increased expression of HSP90B1 in renal cancer cells (Fig. 2C) and ccRCC tissues (Fig. 2D). These results validated the high expression of HSP90B1 in ccRCC as obtained through bioinformatics analysis. Based on these results, 786-O and ACHN, which exhibited relatively higher levels of HSP90B1 expression, were selected for subsequent experiments.

### 3.3 AUY922 exerts an inhibitory effect on renal cancer cells

**Fig. 3:**
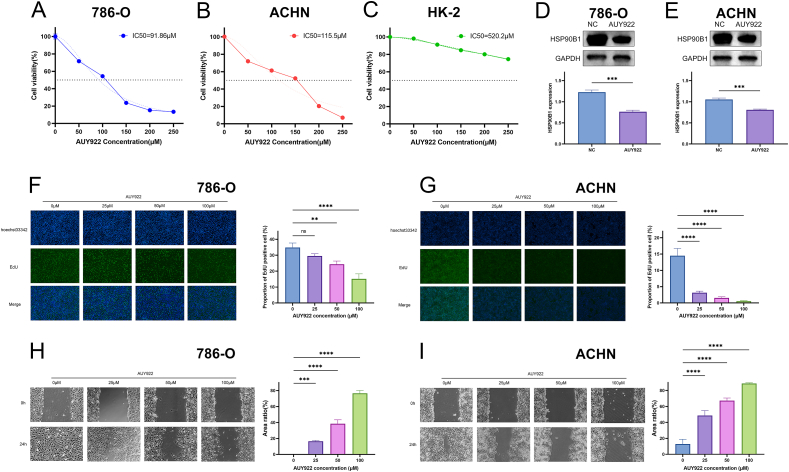
AUY922 exerts an inhibitory effect on renal cancer cells. Cell viability of the cell lines was assessed using the CCK-8 assay: (A) 786-O, (B) ACHN, (C) HK-2; Western blot Images and quantification based on GAPDH served as internal reference of HSP90B1 expression after treating with 50 μM AUY922(The original image is provided in the Supplementary Fig. S2): (D) 786-O, (E) ACHN; AUY922 inhibits the proliferation of ccRCC cells using the EdU assay: (F) 786-O, (G) ACHN. AUY922 inhibits the migration of ccRCC cells were assessed by using the wound healing assay: (H) 786-O, (I) ACHN.

A CCK-8 assay was conducted to ascertain the impact of AUY922 on renal cancer cell activity. The results showed a continuous decrease in cell activity after 24 h of treatment with different concentrations of AUY922. The IC50 was 91.86 μM for the 786-O and 115.5 μM for the ACHN (Fig. 3A and B). Moreover, AUY922 had a minimal cytotoxic effect on HK-2 cell line, with an IC50 of 520.5 μM, almost five times higher than that for 786-O and ACHN (Fig. 3C). Next, we selected the suitable concentration of AUY922 (50 μM) to treat the cells for 24 h. We detected the change in HSP90B1 expression through Western blot analysis. The results showed a significant down-regulation of HSP90B1 expression in 786-O and ACHN after AUY922 treatment (Fig. 3D and E).

The EdU assay was utilized to evaluate the effect of AUY922 on the proliferation of renal cancer cells. Using immunofluorescence analysis, we determined the percentage of EdU-positive cells following a 24-h treatment with AUY922. The results showed that the proportion of EdU-positive cells decreased as the concentration of AUY922 increased, indicating that AUY922 inhibits the proliferation of renal cancer cells (Fig. 3F and G). To investigate the impact of AUY922 on the migration of renal cancer cells, we performed a wound healing assay. The results demonstrated that AUY922 inhibited the migration of renal cancer cells, with increasing potency as the concentration of AUY922 increased (Fig. 3H and I).

### 3.4 Network pharmacology reveals potential mechanism of AUY922 in ccRCC

**Fig. 4:**
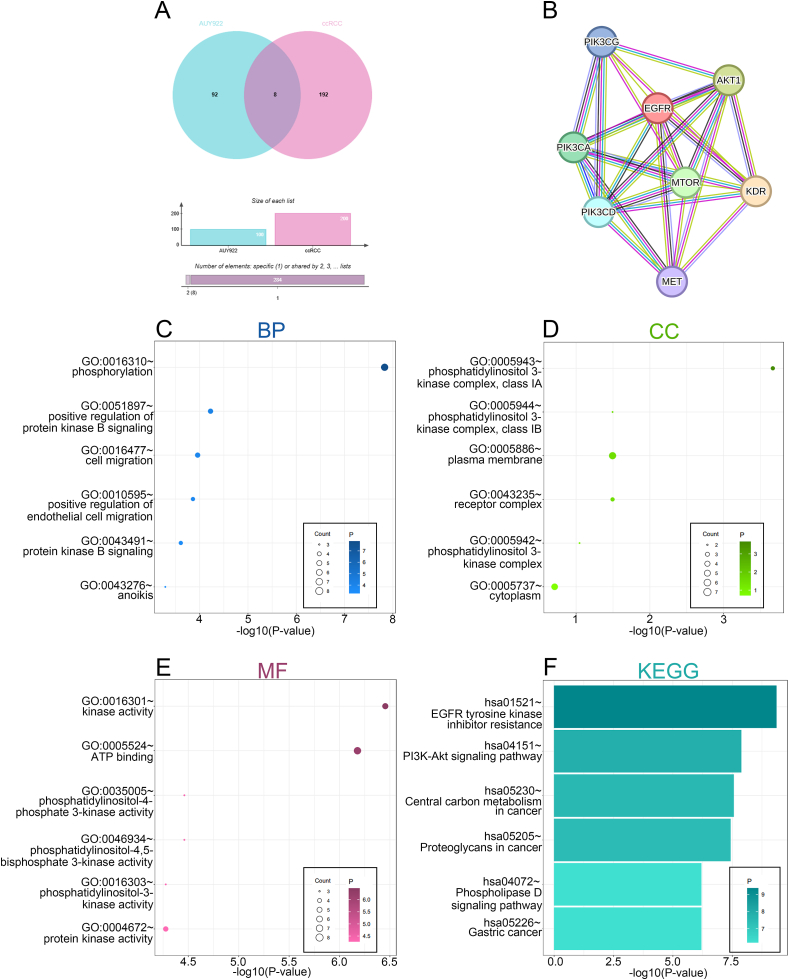
Network pharmacology analysis between AUY922 and ccRCC. (A) Venn diagram of AUY922 target genes and ccRCC disease genes. (B) PPI network of the overlapping genes. (C) BP, (D) CC, (E) MF, (F) KEGG of the overlapped genes.

A Venn diagram was employed to analyze the AUY922 target genes and ccRCC disease genes obtained from databases. This analysis revealed a total of eight genes that were overlapped between the two sets, including MTOR, PIK3CA, KDR, MET, EGFR and others (Fig. 4A, Supplementary Table 1). These genes were then mapped into a PPI network complex (Fig. 4B). Subsequently, a comprehensive functional analysis of the overlapping genes was conducted, focusing on biological processes (BP), cellular components (CC), molecular functions (MF), and KEGG pathways. The findings revealed that the BP associated with the overlapping genes were primarily related to phosphorylation, positive regulation of protein kinase B signaling, cell migration, positive regulation of endothelial cell migration, protein kinase B signaling, and anoikis (Fig. 4C). Additionally, the overlapping genes were significantly present in CC such as the phosphatidylinositol 3-kinase complex, plasma membrane, receptor complex, and so on (Fig. 4D). In terms of MF, the overlapping genes were primarily involved in kinase activity, including kinase activity, ATP binding, protein kinase activity (Fig. 4E). Regarding KEGG pathways, the overlapping genes exerted a considerable influence on pathways including EGFR tyrosine kinase inhibitor resistance, PI3K-Akt signaling pathway, Central carbon metabolism in cancer, and proteoglycans in cancer (Fig. 4F). The preceding analysis demonstrated that AUY922 exhibited a high degree of association with PTK inhibitors, such as sunitinib, in ccRCC.

### 3.5 AUY922 enhances the sensitivity of sunitinib in renal cancer cells

**Fig. 5:**
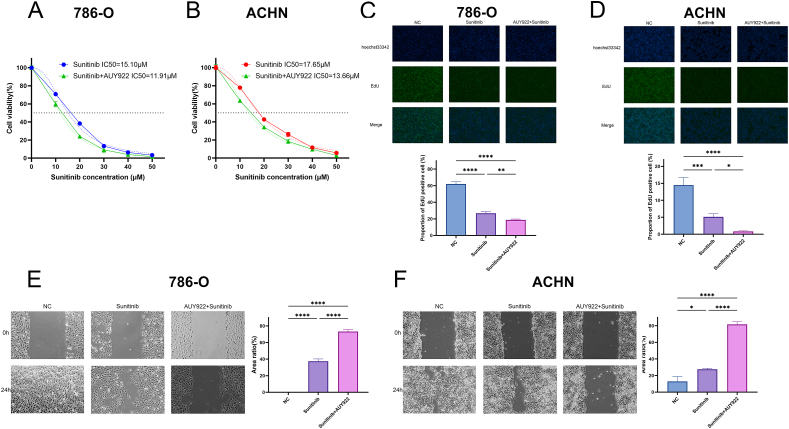
AUY922 enhances the inhibitory effect of sunitinib on renal cancer cells. The IC50 of ccRCC cells for sunitinib was determined using the CCK-8 assay: (A) 786-O, (B) ACHN; The EdU assay was performed to determine proliferation in ccRCC cells treated with sunitinib and sunitinib combine with AUY922: (C) 786-O, (D) ACHN. The wound healing assay was performed to determine migration in ccRCC cells treated with sunitinib and sunitinib combine with AUY922: (E) 786-O, (F) ACHN. NC: Negative Control; Sunitinib: 5 μM sunitinib; Sunitinib + AUY922: 5 μM sunitinib combine with 50 μM AUY922.

Based on the results of the network pharmacology analysis, we subsequently investigated the effect of AUY922 on the sensitivity of ccRCC cells to sunitinib. Using a CCK-8 assay, we found that the addition of 50 μM AUY922, compared to sunitinib alone, reduced the IC50 of 786-O from 15.10 μM to 11.91 μM, and the IC50 of ACHN from 17.65 μM to 13.66 μM. These results suggest that AUY922 enhances the sensitivity of ccRCC cells to sunitinib (Fig. 5A and B).

Subsequently, we employed EdU assays and wound healing assays to investigate the effect of AUY922 on the inhibition of sunitinib on ccRCC cells. The EdU assay results indicated that the proportion of EdU-positive cells significantly decreased after the application of 5 μM sunitinib, from 62.05 ± 2.76 % to 26.79 ± 1.99 % in 786-O and from 14.49 ± 2.26 % to 5.14 ± 0.87 % in ACHN. This proportion further decreased to 18.78 ± 1.02 % in 786-O and 0.82 ± 0.17 % in ACHN after the addition of AUY922. (Fig. 5C and D). The wound healing assay results showed that the wound area significantly increased after sunitinib treatment, with 786-O increasing to 37.43 ± 2.76 % and ACHN increasing to 27.52 ± 1.34 %. Following combined treatment with sunitinib and AUY922, the wound area increased to 73.22 ± 2.19 % in 786-O and 81.56 ± 3.33 % in ACHN (Fig. 5E and F). These results demonstrated that sunitinib inhibits the proliferation and migration of ccRCC cells, and AUY922 further enhances the inhibitory effect of sunitinib on ccRCC cells.

### 3.6 AUY922 targeting HIF-1α/VEGFA/VEGFR pathway

**Fig. 6:**
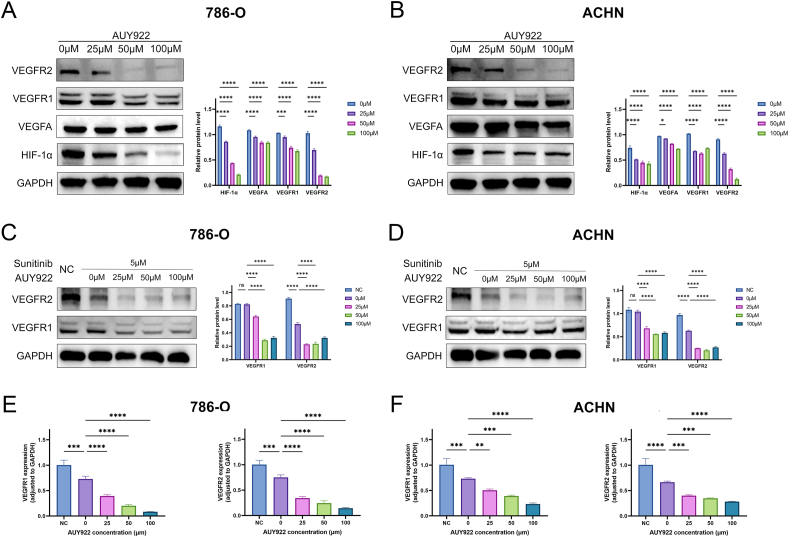
AUY922 targeting HIF-1α/VEGFA/VEGFR pathway. Images and quantification based on GAPDH served as internal reference of Western blot of VEGFR2, VEGFR1; VEGFA and HIF-1α: (A) 786-O, (B) ACHN (The original image is provided in the Supplementary Fig. S3); Images and quantification based on GAPDH served as internal reference of Western blot of VEGFR1 and VEGFR2 after treatment of 5 μM sunitinib and 5 μM sunitinib combine with AUY922: (C) 786-O, (D) ACHN (The original image is provided in the Supplementary Fig. S4). Quantification based on GAPDH served as internal reference of RT-PCR of VEGFR1 and VEGFR2 after of 5 μM sunitinib and 5 μM sunitinib combine with AUY922: (E) 786-O, (F) ACHN. NC: Negative Control.

Using Western blot analysis, we investigated the effect of AUY922 on the HIF-1α/VEGFA/VEGFR pathway by assessing the protein expression levels of HIF-1α, VEGFA, VEGFR1, and VEGFR2 in renal cancer cells. The results showed that treatment with AUY922 led to a reduction in the expression levels of HIF-1α, VEGFA, VEGFR1, and VEGFR2 (Fig. 6A and B). These results suggest that AUY922 specifically targets and inhibits the HIF-1α/VEGFA/VEGFR signaling pathway in renal cancer cells, which may contribute to its therapeutic effects.

To investigate if AUY922 has a synergistic effect with sunitinib, the expression levels of VEGFR were assessed after treatment with sunitinib alone and in combination with AUY922. Western blot results revealed that treatment with sunitinib led to a decrease in the protein expression levels of both VEGFR1 and VEGFR2 in 786-O and ACHN. Additionally, as the combination with AUY922, the expression levels of VEGFR1 and VEGFR2 further decreased (Fig. 6C and D). RT-PCR results also demonstrated that the addition of AUY922 resulted in lower mRNA expression levels of VEGFR1 and VEGFR2 in renal cancer cells compared to treatment with sunitinib alone (Fig. 6E and F).

## 4 Discussion

Sunitinib, a first-line treatment for the treatment of advanced ccRCC, is effective in inhibiting angiogenesis and cell proliferation. However, most patients develop resistance to sunitinib within 15 months of treatment, which is a major challenge in ccRCC treatment [19]. Currently, three main therapeutic strategies for sunitinib-resistant ccRCC are sunitinib com-bination therapy, sequential therapy with VEGFR inhibitors, and anti-cancer vaccines. Among these strategies, combination therapy has been extensively studied [20]. Shi et al. found that combining sunitinib with vincristine could increase sensitivity and decrease resistance to sunitinib in ccRCC patients [21]. Ashaq et al. conducted experimental studies and found that the combination of sunitinib with Hispidulin, a novel natural compound, significantly increased the mortality of ccRCC cells and improved survival in ccRCC mice compared to sunitinib alone [22]. Therefore, exploring new combinations to increase the sensitivity of ccRCC cells to sunitinib is critical to address sunitinib resistance in ccRCC.

The HSP90 family, including Heat Shock Protein 90 Alpha Family Class A Member 1 (HSP90AA1), Heat Shock Protein 90 Alpha Family Class B Member 1 (HSP90AB1), HSP90B1, and TNF Receptor Associated Protein 1 (TRAP1), plays a pivotal part in various cellular processes and in the folding of client proteins involved in cancer development [23]. HSP90B1, a member of the HSP90 family, is abundant in cancer cells and can act as a carrier of tumor antigenic peptides, playing a critical role in tumor antigen presentation and activation of CD8^+^ T lymphocytes [24]. Targeting HSP90B1 has been explored as a potential strategy for tumor suppression. AUY922, a novel HSP90 inhibitor, effectively suppresses HSP90, including HSP90B1. In addition to investigating the direct inhibitory effect of AUY922 on tumors, studies have also focused on combining AUY922 with chemotherapeutic agents to enhance tumor chemosensitivity. For example, AUY922 in combination with cytarabine shows significant synergistic anti-leukemic activity in vivo [25]. The combination of AUY922 with lapatinib improves the sensitivity of drug-resistant gastric cancer to lapatinib [26].

Based on the above concepts, we initially employed bioinformatics analysis to predict the expression of HSP90B1 in ccRCC. Pan-cancer analysis, along with data from the GEO, TCGA, and GTEx databases, indicated that HSP90B1 is highly expressed in ccRCC tissues. Consequently, we further validated these findings using Western blotting and RT-PCR. The results showed that HSP90B1 expression was significantly elevated in ccRCC tissues and cell lines compared to adjacent non-cancerous tissues and normal cell lines, although the increase was less pronounced in the Caki-1 cell line, possibly due to cellular heterogeneity. We subsequently analyzed the relationship between HSP90B1 expression and the clinicopathological features of ccRCC. Interestingly, high HSP90B1 expression was significantly associated with T4 and N1 stages, which are indicative of tumor metastasis and progression [8], but showed no significant difference with WHO grading, which is unrelated to metastasis, or with the M stage, which indicates that metastasis has already occurred. These findings suggest that ccRCC patients with high HSP90B1 expression may be prone to tumor metastasis and rapid progression. This insight could potentially guide personalized prevention and treatment strategies through genetic testing in clinical settings for ccRCC patients. Subsequently, we investigated the impact of AUY922 on ccRCC cell lines 786-O and ACHN by downregulating HSP90B1. The experimental results demonstrated that AUY922 treatment led to a reduction in HSP90B1 expression levels in both 786-O and ACHN, resulting in significantly inhibited cell viability. In contrast, the viability of HK-2 cells was not notably affected. Further EdU assays and wound healing assays indicated that AUY922 inhibited the proliferation and migration of ccRCC cells in a dose-dependent manner.

Network pharmacology is a method of predicting drug-disease relationships by using databases to obtain information on genes and proteins associated with drugs and diseases. It is now widely used in various fields, including new drug development and drug-targeted therapies [27]. We performed network pharmacological prediction of AUY922 and ccRCC by utilizing relevant databases and found that the mechanism of AUY922 in ccRCC was associated with RTK inhibitors. Interestingly, sunitinib is a classical RTK inhibitor, and its interaction with AUY922 in ccRCC aroused our great interest. Based on the results of network pharmacology analysis, we investigated the effect of AUY922 on the sensitivity of ccRCC cells to sunitinib. The CCK-8 assay comparing IC50 values revealed that AUY922 increased the sensitivity of ccRCC cells to sunitinib. Subsequent EdU assays and wound healing assays further demonstrated that AUY922 enhanced the inhibitory effect of sunitinib on ccRCC cells.

Hypoxia-inducible factor 1α (HIF-1α), a transcription factor encoded by the HIF1A gene and plays a critical role in cellular responses to hypoxia, has been found to be closely associated with HSP90, as HIF-1α binds to HSP90 in the cytoplasm and increases its stability. Inhibiting the binding of HSP90 to HIF-1α with HSP90 inhibitors can destabilize and degrade HIF-1α, thereby inhibiting VEGFR production [28]. In addition, the signaling pathway involving HIF-1α, its downstream factor VEGFA, and its receptor VEGFR has received considerable attention for its role in promoting tumor growth and metastasis. Chen et al. discovered that bone morphogenetic protein 9 (BMP9) promotes angiogenesis in hepatocellular carcinoma by activating the HIF-1α/VEGFA signaling pathway, and inhibiting BMP9 to regulate the HIF-1α/VEGFA signaling pathway may be a potential treatment option for advanced hepatocellular carcinoma [29]. Huang et al. found that sesamin inhibited colorectal cancer angiogenesis by targeting the NF-κB/HIF-1α/VEGFA signaling pathway, suggesting its potential in the prevention and treatment of colorectal cancer [30]. While many ccRCC cases are sporadic, evidence suggests that hereditary ccRCC is primarily caused by loss or silencing of the VHL gene, leading to activation of HIF-1α activation and subsequent upregulation of pro-angiogenic factors such as VEGF [31]. Furthermore, VEGFR serves as a molecular target for sunitinib. Therefore, we explored whether HIF-1α/VEGFA/VEGFR signaling pathway influences the process by which AUY922 in-creases renal cancer sensitivity to sunitinib. Western blotting revealed a significant de-crease in the expression levels of HIF-1α, VEGFA, VEGFR1 and VEGFR2 after AUY922 treatment. And after combining AUY922 and sunitinib, both Western blot and RT-PCR suggested that AUY922 enhanced the targeted inhibitory effect of sunitinib on VEGFR1 and VEGFR2.

Our study is the first to identify that AUY922 can enhance the sensitivity of ccRCC to sunitinib. AUY922 not only has an inhibitory effect on ccRCC cells, but also enhances the inhibitory effect of sunitinib on ccRCC cells. Additionally, our research is the first to explore the mechanism of AUY922 in ccRCC, demonstrating that it targets the HIF-1α/VEGFA/VEGFR pathway by inhibiting HSP90B1.

However, our study has some limitations. Firstly, our study was only validated by in vitro experiments with renal cancer cells, and further in vivo experiments are necessary in the future. Secondly, although our study demonstrated that AUY922 increased the sensitivity of ccRCC to sunitinib, more in-depth exploration of the effect of AUY922 on increasing the sensitivity of drug-resistant ccRCC patients to sunitinib is needed in the future. There is still a long way to go before this regimen can enter clinical trials.

## 5 Conclusion

In summary, using bioinformatics, Network pharmacology and in vitro experiments, our study demonstrates that AUY922 had an inhibitory effect on ccRCC and improved the sensitivity of ccRCC to sunitinib. Furthermore, it provides novel insights into the development of strategies to combat sunitinib-resistant ccRCC.

## Ethics approval and consent to participate

This study was reviewed and approved by the Ethics Committee of Tongren Hospital, Shanghai Jiao Tong University School of Medicine with the approval number:2019-099-01, dated: 2019/12/01. All patient provided written informed consent to participate in the study and for their data to be published.

## Funding

This research was funded by grants from the 10.13039/501100001809National Natural Science Foundation of China (No. 82172809), Research Fund of Shanghai Tongren Hospital, Shanghai Jiaotong University School of Medicine (No. TRKYRC-xx202205).

## Data availability statement

The data that support the findings of this study are available from the corresponding author, upon reasonable request.

## CRediT authorship contribution statement

**Zixuan Chen:** Writing – original draft, Visualization, Conceptualization. **Xing Jia:** Validation, Resources. **Yuesong Cai:** Visualization, Validation. **Ya Song:** Software. **Yanjun Tong:** Resources. **Sheng Cheng:** Writing – review & editing, Supervision, Investigation, Funding acquisition. **Min Liu:** Writing – review & editing, Supervision, Project administration, Funding acquisition, Conceptualization.

## Declaration of competing interest

The authors declare that they have no known competing financial interests or personal relationships that could have appeared to influence the work reported in this paper.
