# Supplementary material for: AUY922 improves sensitivity to sunitinib in clear cell renal cell carcinoma based on network pharmacology and in vitro experiments
Source: Heliyon. 2024 Jul 18;10(14):e34834. doi: 10.1016/j.heliyon.2024.e34834 (PMC11324986; doi:10.1016/j.heliyon.2024.e34834)
Supplement: Multimedia component 1 [file mmc1.docx]

**Supplementary Table S1. Primers used for RT-PCR analysis.**

| Names | Sequence |
| --- | --- |
| F-HSP90B1 | GTCAAGGGTGTGGTGGACTC |
| R-HSP90B1 | TGTCCAGCGTTTTACGAACAAG |
| F-VEGFR2 | TGACAACCAGACGGACAGTG |
| R-VEGFR2 | GGGCACCATTCCACCAAAAG |
| F-VEGFR1 | ATACCTCACTGTTCAAGGAACC |
| R-VEGFR1 | ATAGGAGCCAGAAGAGAGTCG |
| F-GAPDH | AGGTCGGAGTCAACGGATTT |
| R-GAPDH | GCCATGGGTGGAATCATATTGG |

**Supplementary Table S2. The overlapped genes of AUY922 target genes and RCC disease genes.**

| genes | Gene description |
| --- | --- |
| MTOR | Mechanistic Target Of Rapamycin Kinase |
| PIK3CA | Phosphatidylinositol-4,5-Bisphosphate 3-Kinase Catalytic Subunit Alpha |
| KDR | Kinase Insert Domain Receptor |
| MET | MET Proto-Oncogene, Receptor Tyrosine Kinase |
| EGFR | Epidermal Growth Factor Receptor |
| PIK3CD | Phosphatidylinositol-4,5-Bisphosphate 3-Kinase Catalytic Subunit Delta |
| AKT1 | AKT Serine/Threonine Kinase 1 |
| PIK3CG | Phosphatidylinositol-4,5-Bisphosphate 3-Kinase Catalytic Subunit Gamma |

**Supplementary Fig. S1**

**
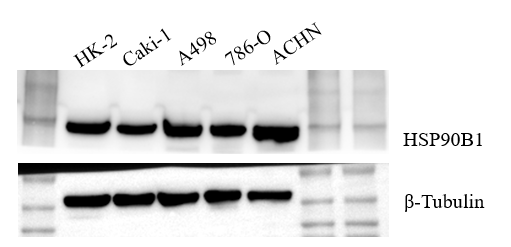
**

**Supplementary Figure 1. Uncropped western blots used for Figure 2A.**

**Supplementary Fig. S2**

**
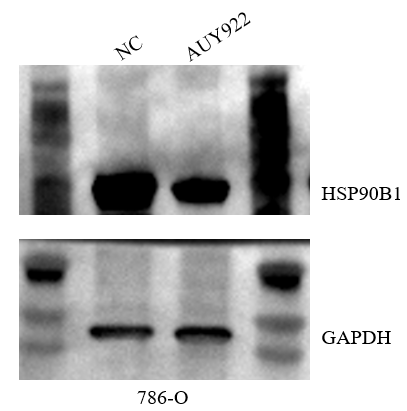

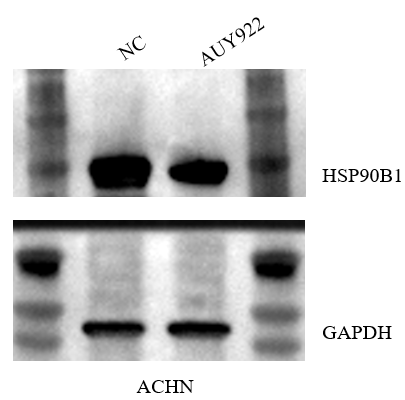
**

**Supplementary Figure 2. Uncropped western blots used for Figure 2D and 2E.**

**Supplementary Fig. S3**

**
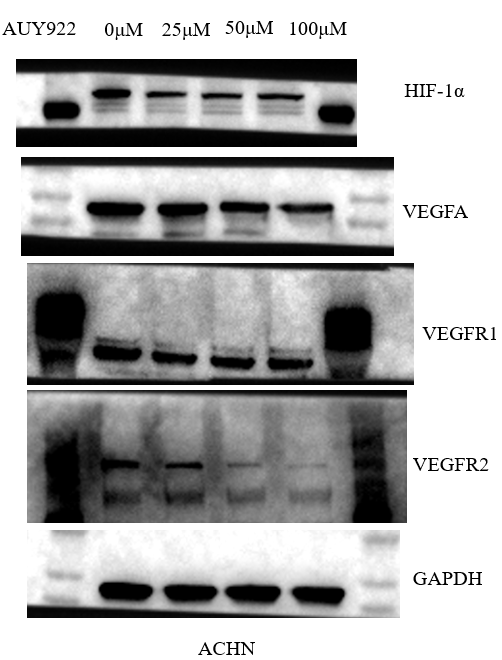
**

**
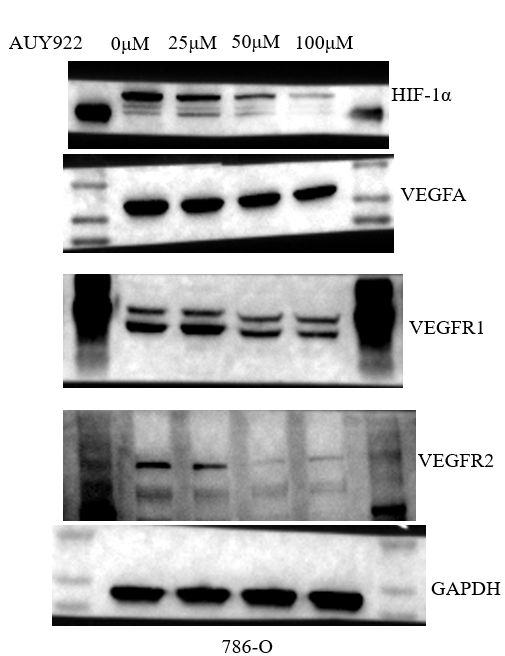
**

**Supplementary Figure 3. Uncropped western blots used for Figure 6A and 6B.**

**Supplementary Fig. S4**

**
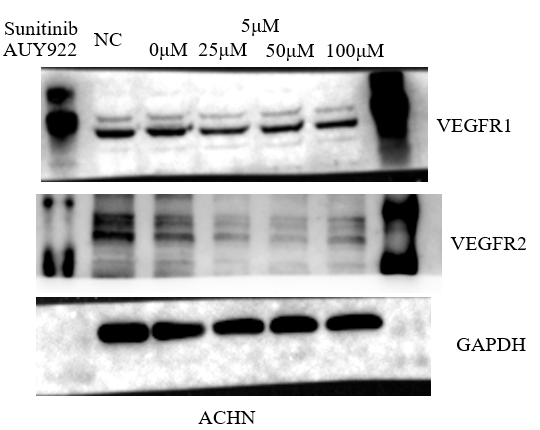
**

**
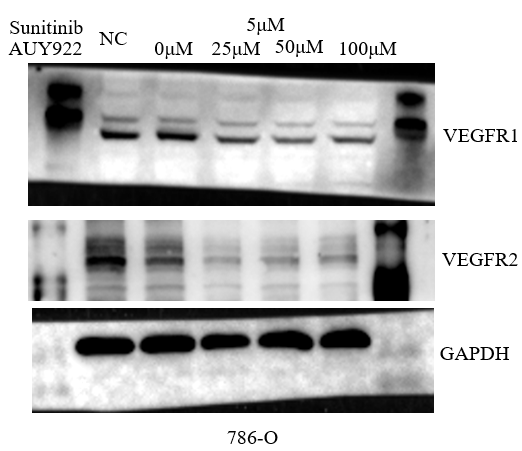
**

**Supplementary Figure 4. Uncropped western blots used for Figure 6C and 6D.**
